# Choice and Uncertainty Clog the Bottleneck of Central Processing

**DOI:** 10.1371/journal.pbio.0040244

**Published:** 2006-06-27

**Authors:** Jami Milton Dantzker

In today's fast-paced computer age, multitasking has become routine. But even the most skillful multitaskers can't “think” about two tasks at exactly the same time. Psychologists and neuroscientists study parallel processing in the brain by modeling information as traveling sequentially through three processing stages categorized as perceptual, decision (the central black box of the mind), and motor output. A long-standing hypothesis holds that the central decision stage is a passive bottleneck, where two competing tasks are handled on a first-come, first-served basis. In this model, the second task engages only after the first has passed through the bottleneck and does not slow down the reaction time on the first task, even when the second is presented rapidly after the first, also know as short stimulus onset asynchrony (SOA).

Growing evidence questions whether the passive bottleneck model fully captures the complexity of task-switching dynamics. For example, last year in *PLoS Biology*, researchers Mariano Sigman and Stanislas Dehaene reported that, during dual-tasks, response times to the first task lengthened compared with performing the first task alone, independently of SOA. They proposed that since response order was dictated in the experiment, this additional processing time resulted from the participant's brain dictating—like issuing an executive order—which task to perform first, in keeping with the experimental instructions.

In a new study, the researchers employed the same dual-tasks used in their previous study—number comparison and tone discrimination (link to previous synopsis: http://biology.plosjournals.org/perlserv/?request=get-document&doi=10.1371/journal.pbio.0030084). But this time they made task order unpredictable and the subjects decided which task to perform first. With choice, it turns out, task order and reaction times vary in a manner suggesting that uncertainty comes at a cost, and that active processes during the perceptual and central stages are required to decide when to engage and disengage each task.

As before, the number and tone tasks involved making simple discriminations as accurately and quickly as possible. Subjects decided whether a number was greater or less than 45. Cognitive challenge resulted from varying the task by notation (Arabic digits or words) and distance (numerical distance between a presented number and 45). The tone task (is a tone high or low pitch?) did not vary, except for the order of presentation and timing relative to the number task. Subjects responded to each task by pressing a key (visual with the right hand and auditory with the left).

Subjects tended to respond to the number task first, but presentation order also significantly affected response order. When the two tasks were presented in close succession (SOA), the choice is harder. Thus, as in their first experiment, the researchers expected that subjects would perform the first task more slowly in the dual-task; and, contrary to the first experiment, they expected timing to increase for small SOAs when there is a conflict on which task to perform first.

It turns out that Sigman and Dehaene made several key observations that were inconsistent with the passive bottleneck model, especially during small SOAs. The first departure was revealed by the observation that the reaction time to the first task increased as SOA decreased. The researchers argue that this indicates a stochastic, “bottom-up” perceptual mechanism—that is, from lower to higher cognitive processes—that weighs information before devoting central resources to a task. They call this a “task setting” stage, and propose that, as SOA gets shorter, the second task interferes with the first, and task setting becomes more difficult.

To explain the remaining departures from the passive bottleneck model, the researchers propose a “task disengagement” stage that has a relatively fixed duration. In this case, they argue, while the second task can enter the central processing stage soon after completing the first task, the motor stage for the second task does not engage until the previous processing mode is complete. In their previous study, Sigman and Dehaene found that manipulating the distance in the number task taxed the central stage. In this study, however, this relationship no longer held when the SOA was less than 400 milliseconds, suggesting the task disengagement process is instead the limiting factor at work. Also, when comparing the relationship between response times and SOA, they saw less variation in response times during the dual-task than in each individual task, suggesting that the response timing was relatively locked.

The researchers acknowledge that their model may not be the only possible explanation, nor may it apply to more-complex tasks. However, by systematically testing the predictions from the passive bottleneck model, they confirmed that more-active central processes are necessary to understand the cognitive architecture of multitasking, and argue that this new model leads to new testable predictions. Moreover, their model synthesizes prior work on task-switching costs, bottlenecks, and hierarchies of cognitive architecture, thus providing a solid framework for future studies. Based on this work, the researchers hope to integrate their findings with functional brain images to map the different stages of information processing to specific brain areas.

## 

**Figure:**
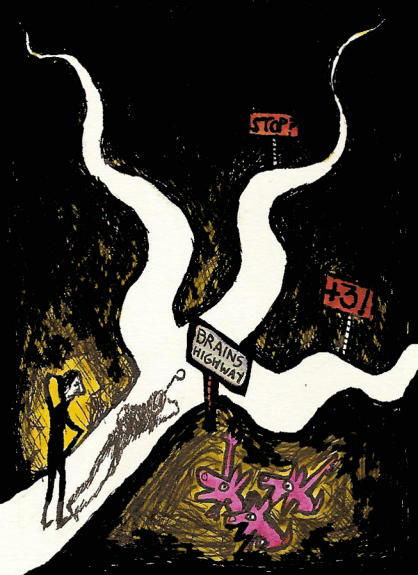
When faced with a decision to perform multiple, competing tasks, neural processes put constraints on which task to perform first and how quickly to do it.

